# Contamination of Dentin with Hemostatic Agents – Is EDTA a Valuable Decontaminant before Using a Self-etch Universal Adhesive?

**DOI:** 10.3290/j.jad.b3441525

**Published:** 2022-09-28

**Authors:** Carolin Anne Mempel, Silke Jacker-Guhr, Anne-Katrin Lührs

**Affiliations:** a Doctoral Student, Department of Conservative Dentistry, Periodontology and Preventive Dentistry, Hannover Medical School, Hannover, Germany. Wrote the manuscript, prepared the specimens, performed the μTBS test, performed the statistical analysis in fulfilment of the requirements for the degree “Dr. med. dent.”; b Senior Lecturer, Department of Conservative Dentistry, Periodontology and Preventive Dentistry, Hannover Medical School, Hannover, Germany. Study concept, proofread the manuscript.; c Senior Lecturer and Professor, Department of Conservative Dentistry, Periodontology and Preventive Dentistry, Hannover Medical School, Hannover, Germany. Research idea, study concept, performed the statistical analysis, co-wrote paper, proofread the manuscript.; * Contributed equally to this paper.

**Keywords:** universal adhesives, self-etch, ethylenediaminetetraacetic acid, hemostatic agents, aging, microtensile bond strength.

## Abstract

**Purpose:**

To investigate the effects of dentin decontamination procedures with ethylenediaminetetraacetic acid (EDTA) after contamination with two hemostatic agents, ViscoStat (VS) and ViscoStat Clear (VSC), on the microtensile bond strength (μTBS) of two different universal adhesives, before and after thermocycling (TC).

**Materials and Methods:**

Dentin surfaces of 100 human caries-free molars were either contaminated with one of the hemostatic agents or contaminated and then decontaminated with EDTA before the universal adhesives Scotchbond Universal Adhesive (SBU) or Prime & Bond Active (PBA) were applied in self-etch mode. Composite buildups were made and the teeth were sectioned into sticks (n = 90). Half of them immediately underwent the μTBS test, the other half after aging via TC. The data were statistically analyzed using Welch’s ANOVA and the Games-Howell post-hoc test (p < 0.05).

**Results:**

Significant differences were observed between the groups (p < 0.001). When the dentin surface was contaminated with VSC, TC significantly reduced the mean bond strength, regardless of the universal adhesive. Decontamination with EDTA showed a significant decrease in bond strength after VS contamination and SBU application. The fracture analysis showed mainly adhesive fractures (78.8%) in all test groups.

**Conclusion:**

As EDTA application did not significantly increase the μTBS of either universal adhesive in self-etch mode in-vitro, it cannot be recommended as a decontaminant.

The bond strength between adhesives and the respective tooth surface is negatively influenced by numerous factors, including a wide variety of contaminants, eg, blood and saliva.^1,16,40^ Contamination can also occur by hemostatic agents, which are helpful for managing bleeding in deep cavities, but also pose the risk of interfering with adhesive procedures.^2,18,25^

In case of blood contamination, the influence of the protein content and the presence of fibrinogen and platelets seems to be decisive. Together, these molecules form a barrier in the form of a thin film on the dentin surface, which hampers the penetration of the adhesive into the dentinal tubules as well as the wetting of the dentin surface, which are relevant for the bond.^1^ Such a blockage adversely modifies the configuration of the hybrid layer^32^ by preventing the formation of complete microtags. Since these are indispensable for the micromechanical bond of the adhesive to dentin,^38^ this in turn leads to a reduction in bond strength of 30%–70%.^40^ To avoid this type of contamination, the use of rubber-dam is recommended.^31^ However, in several clinical situations, such as deep class-V or subgingival cavities, the use of rubber-dam is simply not expedient, as either the gingiva has previously been irritated by the preparation or the cavity depth does not allow proper isolation, which in turn causes contamination of the tooth surface by blood. In those cases, the use of a hemostatic agent can be beneficial for controlling gingival bleeding and the efflux of gingival fluid. As a consequence, a rather dry working field for adhesive procedures can be created in situations where isolation by rubber-dam is not possible, by using individualized matrix bands or a retraction cord in combination with hemostatic agents.^21,22^ For this purpose, astringents, which are solutions containing aluminum chloride, aluminum sulfate, or ferric sulfate, can be used. They are acidic and have pH values in the range of 0.7–3.0.^20,39^ The aim of hemostasis in this particular process is to close the capillary openings by coagulum plugs.^14,25^

Universal adhesives, also known as multimode adhesives, can be applied in either self-etch, selective enamel etch or etch-and-rinse mode.^38^ Most universal adhesives contain the monomer 10-MDP (methacryloyloxydecyl dihydrogen phosphate), which stabilizes dentin-adhesive-composite interfaces by “nano-layering”.^42^ During self-etch and selective enamel-etch procedures, a chemical bond to the calcium of the dentin can be established.^41^ The durability of a composite restoration depends, among other things, on the correct application of the adhesive. For instance, universal adhesives benefit from an “active” application on the dentin surface by using a microbrush in a scrubbing motion.^24^

Various studies have documented the negative influence of dentin contamination by astringents such as hemostatic agents on the adhesive bond.^2,18,25,28^ More precisely, the bond strength to dentin after contamination with a 25% aluminum-chloride solution was significantly lower than that of the control groups.^18^ The remnants of the contaminating aluminum-chloride agent leave an acid-resistant layer on the dentin surface, which cannot be completely removed by the monomer of the universal adhesive in self-etch mode.^2^

Because contamination of dentin before the use of an adhesive might interfere with bond strength, especially when used in self-etch mode, various “decontamination” protocols have been described in the literature,^25^ for example water rinsing, chlorhexidine, etching with phosphoric acid, or ethylenediaminetetraacetic acid (ETDA).^10^

EDTA is a chelating agent which can demineralize the tooth surface, as it is able to form a chelate complex with Ca^2+^. The degree of protonation depends on the pH value of the solution and the application time.^29,35^ EDTA solutions are available in various concentrations and at neutral or alkaline pH values. A 17% EDTA solution with a pH of 7.5 has proven to be most effective during demineralization of the root canal dentin.^29^ In addition, EDTA seems to be able to remove the smear layer shortly after its application to root dentin.^29^ When EDTA was used as a decontaminant after contamination of dentin surfaces by a hemostatic agent, it was able to restore the bond strength of a self-etch adhesive to the level of adhesion measured for non-contaminated dentin.^2^

Since contamination by hemostatic agents may interfere with the bond strength of universal adhesives to dentin, the aim of the study was to determine whether decontamination with EDTA after surface contamination with two different hemostatic agents is an effective method for restoring the bond strength.

The following null hypotheses were set forth:

- The microtensile bond strength of the universal adhesives used in self-etch application is not significantly different.
- The microtensile bond strength is not influenced by surface contamination with hemostatic agents.
- A “decontamination” with EDTA solution does not affect the microtensile bond strength.
- Aging by thermocycling does not influence the microtensile bond strength.

## MATERIALS AND METHODS

In this in-vitro study, the effects of two different hemostatic agents (ViscoStat and ViscoStat Clear, Ultradent; South Jordan, UT, USA) on the dentin bond strength of two universal adhesives (Scotchbond Universal Adhesive, 3M Oral Care; St Paul, MN, USA, and Prime & Bond Active, Dentsply Sirona; Konstanz, Germany) were investigated with and without surface decontamination using a 20% EDTA solution (CALCINASE EDTA solution, lege artis; Dettenhausen, Germany). One hundred human caries- and restoration-free molars were stored in a 0.5% chloramine solution in a refrigerator at 4°C and used within 6 months after extraction. The use of extracted teeth for bond strength testing was approved by the Ethics Commission of the Hannover Medical School (no. 2092-2013). All materials used in this study and their application are shown in Table 1.

**Table 1:** Materials and their application based on manufacturer’s instructions

Material	Manufacturer	Manufacturer’s instructions with their adaption to the study design	Lot No.	Expiration date
Scotchbond Universal Adhesive (SBU, pH 2.7)	3M Oral Care; St Paul, MN, USA	Active application on dentin surface for 20 s with microbrush, gentle air stream for approximately 5 s, until no more liquid movement is visible, light curing for 10 s.	6703873	2021-12-06
Prime & Bond Active Universal Adhesive (PBA, pH 2.5)	Dentsply Sirona; Konstanz, Germany	Active application on dentin surface for 20 s with microbrush, gentle air stream blowing for approximately 5 s, until no more liquid movement is visible, light curing for 10 s.	1911000710	2021-08-31
CALCINASE EDTA-solution (EDTA, 20% sodium edetate, pH 8.4)	lege artis; Dettenhausen, Germany	Application to the dentin surface by microbrush, no active movement, but surface was kept moist, exposure time 2 min, water rinsing of the surface for 10 s.	0840819	2022-08
ViscoStat 20% Ferric sulfate (VS, pH 1.0)	Ultradent; South Jordan, UT, USA	Application to the dentin surface by microbrush, reaction time 2 min, rinsing of the surface with a strong air-water jet for 30 s.	BHZAY	2023-10-31
ViscoStat Clear 25% Aluminum chloride (VSC)	Ultradent	Application to the dentin surface by microbrush, reaction time 2 min, rinsing of the surface with a strong air-water jet for 30 s.	BHP82	2023-04-30
3M Filtek Universal Restorative	3M Oral Care	Application of 3 individual layers of a maximum of 2 mm thick each, light curing for 20 s (> 1000 mW/cm^2^) from the occlusal aspect. After the last layer, light curing for 20 s from all 4 lateral surfaces.	NA82277	2022-05-28

The teeth were randomly divided into 10 main groups with 10 teeth each (n = 10), resulting in 90 test specimens (n = 90) per group: control groups (application of the adhesive in self-etch mode only), contamination groups (ViscoStat and ViscoStat Clear) and decontamination groups (ViscoStat and ViscoStat Clear with EDTA). Half of the specimens per tooth from each main group (n=45) were tested after 24 h, the other half after thermocycling (TC, for 15,000 cycles). In total, 20 groups were part of the experimental design. During the experiments, the teeth were kept moist at all times by storage in demineralized water. All control and experimental groups are shown in Table 2.

**Table 2:** Control groups (1-4) and experimental groups (5-20) with applied adhesive, contamination by a hemostatic agent, decontamination, thermocycling and their respective coding

Group	Adhesive (SBU/PBA)	Hemostatic agent (VS/VSC)	Decontamination (yes/no)	TC: thermocycling (yes/no)	Coding
1	SBU	-	-	No	SBU
2	SBU	-	-	Yes	SBU*
3	PBA	-	-	No	PBA
4	PBA	-	-	Yes	PBA*
5	SBU	VS	Yes	No	SBU_VS_EDTA
6	SBU	VS	Yes	Yes	SBU_VS_EDTA*
7	PBA	VS	Yes	No	PBA_VS_EDTA
8	PBA	VS	Yes	Yes	PBA_VS_EDTA*
9	SBU	VS	No	No	SBU_VS
10	SBU	VS	No	Yes	SBU_VS*
11	PBA	VS	No	No	PBA_VS
12	PBA	VS	No	Yes	PBA_VS*
13	SBU	VSC	Yes	No	SBU_VSC_EDTA
14	SBU	VSC	Yes	Yes	SBU_VSC_EDTA*
15	PBA	VSC	Yes	No	PBA_VSC_EDTA
16	PBA	VSC	Yes	Yes	PBA_VSC_EDTA*
17	SBU	VSC	No	No	SBU_VSC
18	SBU	VSC	No	Yes	SBU_VSC*
19	PBA	VSC	No	No	PBA_VSC
20	PBA	VSC	No	Yes	PBA_VSC*

*Post-thermocycling. SBU: Scotchbond Universal; PBA: Prime & Bond Active; VS: ViscoStat; VSC: ViscoStat Clear.

### Specimen Preparation

After removal of debris, all teeth were embedded in gypsum parallel to the tooth axis. The occlusal part of the crown was removed under water cooling at perpendicular to the tooth axis using a low speed saw (IsoMet Low Speed Saw, Buehler; Lake Bluff, Illinois, USA). The exposed dentin surface was roughened with moistened sandpaper (600-grit SiC grinding paper, Buehler; Lake Bluff, IL, USA) to create a standardized smear layer. The dentin surfaces were rubbed in circular movements five times to the right and five times to the left over the sandpaper. Afterwards, all specimen surfaces were visually examined for pulpal exposure or residual enamel.

In the control groups, the universal adhesives were actively applied in self-etch mode (Table 1) to the dentin surface for 20 s with a microbrush, then dried by air blowing for 5 s and polymerized for 10 s. No contamination with the hemostatic agents was carried out.

In the contamination groups, the dentin surface was first contaminated with one of the hemostatic agents. After an exposure time of 2 min, the surface was rinsed with an air-water jet for 30 s and then carefully air dried (but not overdried). Then, the respective adhesive was applied according to the manufacturer’s instructions in self-etch mode.

In the decontamination groups, a 20% EDTA solution was applied to the dentin (after contamination with the hemostatic agents) for decontamination. During EDTA application, the surface was kept moist for 2 min with a microbrush. Then, the dentin surface was rinsed with water for 10 s and carefully air dried before the universal adhesive was applied.

In all groups, the adhesive was polymerized for 10 s with a light intensity of >1000 mW/cm^2^ using an LED curing unit (Bluephase, Ivoclar Vivadent; Schaan, Liechtenstein). The light output was checked with a radiometer (Bluephase Meter, Ivoclar Vivadent) before each test cycle to ensure sufficient light output and identical conditions for all samples. The teeth were then built up with a nanofilled composite (3M Filtek Universal Restorative, 3M Oral Care; St Paul, MN, USA), applied in three separate layers of 2 mm thickness maximum. Each layer was light cured from the top surface for 20 s (light intensity >1000 mW/cm^2^). After polymerization of the last layer, further light curing was carried out for 20 s from all four lateral surfaces. The total curing time for each tooth was 140 s.

Subsequently, 4 cuts per tooth on the x- and y-axes were made with a computer-controlled saw (IsoMet High Speed Pro, Buehler; Lake Bluff, IL, USA), which resulted in 9 sticks per tooth (total: 90 sticks in the main group [10 teeth]). Half of the sticks (n = 45) were stored for 24 h in an incubator at 37°C in demineralized water and then subjected to the microtensile bond strength test (μTBS). The other half of the sticks (n = 45) underwent thermocycling (TC, 15,000 cycles, dwell time 30 s, transfer time 10 s, 5°C/55°C). Assuming that 10,000 thermal cycles correspond to one year of in-vivo exposure,^15^ 15,000 cycles are equivalent to 1.5 years of intraoral dwell time for Scotchbond Universal and Prime & Bond Active. This is a time period during which aging effects might appear.

The μTBS was determined with a universal testing machine (MTD-500 plus, SD Mechatronik; Feldkirchen-Westerham, Germany) at a crosshead speed of 1 mm/min. Before testing, all sticks were carefully measured with a caliper (depth x width in mm), and the bonded area was calculated in mm^2^. To detemine the μTBS, the sticks were glued to self-aligning sample holders (SD Mechatronik) of the universal testing machine with cyanoacrylate adhesive (Roxolid Aktiv-X, MEM Bauchemie; Leer, Germany) and then loaded in tension until failure occurred. For calculation of the bond strength (MPa), the maximum force (N) per stick was recorded and divided by the bond area (mm^2^). Sticks which fractured due to manipulation errors were excluded from the statistical analyses; sticks which fractured during aging or cutting were included (as “zero bond”), setting their bond strength as half of the lowest value (in MPa) of the corresponding group.^3^

To determine the location of the fracture, light microscopy (Stemi SV 6, Carl Zeiss; Jena, Germany) at 50X magnification was used. Fractures were divided into adhesive, cohesive in dentin, cohesive in composite, and mixed failures. Fractures that occurred at the interface or at a short distance (≤ 2 mm) into dentin or composite were included in the analyses. Fractures at a greater distance (≥ 2 mm) from the interface were not statistically evaluated.

### Statistical Analysis

The data were tested for normal distribution using the Kolmogorov-Smirnov test. The subsequent statistical analysis of the bond strength was carried out by Welch’s ANOVA and the Games-Howell post-hoc test at a significance level of α < 0.05. The fracture patterns were analyzed using the chi-squared test (SPSS Version 26.0, IBM; Armonk, NY, USA).

## RESULTS

Welch’s ANOVA showed significant differences between the control and test groups (p < 0.001).

### Microtensile Bond Strength (µTBS) of Universal Adhesives Applied in Self-etch Mode to Uncontaminated Dentin

**Fig 1:**
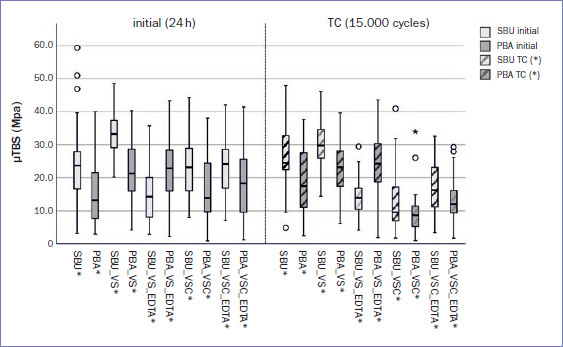
Boxplot of test results (microtensile bond strength in MPa). The median value is represented by the horizontal line within each box, outliners are marked as circles, extreme outliers as asterisks.

In the control groups, where the dentin surfaces were not contaminated, no significant differences were present before TC (SBU vs PBA: p = 0.140). After aging, the mean bond strengths of SBU were significantly higher than those of PBA (SBU* vs PBA*: p = 0.027, Table 3 and Fig 1).

**Table 3:** Results of the µTBS test before and after aging by TC with mean values and standard deviations (in MPa), total number of sticks, zero bonds, and samples excluded from statistical analyses

Group	24-h water storage in an incubator	n/zero bonds/samples excluded from statistics	Aging by TC for 15,000 cycles	n/zero bonds/samples excluded from statistics
SBU	23.7 (± 11.2)adA	41/1/4	26.8 (± 9.5)aA	45/0/0
PBA	16.0 (± 10.6)acA	43/1/2	18.9 (± 9.7)befgA	44/0/1
SBU_VS	33.1 (± 6.5)bA	45/0/0	29.7 (± 8.3)aA	45/0/0
PBA_VS	21.9 (± 8.1)adA	45/0/0	22.3 (± 7.7)adgA	44/0/1
SBU_VS_EDTA	15.3 (± 8.5)cA	45/0/0	14.2 (± 5.4)beA	45/0/0
PBA_VS_EDTA	22.1 (± 9.6)acdA	45/0/0	24.6 (± 8.4)afA	45/0/0
SBU_VSC	23.2 (± 9.5)aA	45/0/0	12.1 (± 8.1)beB	45/1/0
PBA_VSC	16.8 (± 9.6)acdA	45/2/0	8.9 (± 6.3)ceB	45/4/0
SBU_VSC_EDTA	23.6 (± 8.8)adA	45/0/0	17.8 (± 8.1)begA	45/0/0
PBA_VSC_EDTA	19.0 (± 11.0)acdA	45/1/0	13.5 (± 6.7)eA	45/1/0

Groups with the same lowercase (column) or uppercase letter (row) are not significantly different. SBU: Scotchbond Universal; PBA: Prime & Bond Active; VS: ViscoStat; VSC: ViscoStat Clear; TC: thermocycling.

### Influence of Contamination on µTBS

In the case of the universal adhesive SBU, the contamination of the dentin surface with ferric sulfate (VS) initially resulted in a significant increase in bond strength (SBU vs SBU_VS: p = 0.002). After thermocycling, this difference was no longer detectable (SBU* vs SBU_VS*: p = 0.990). When contamination with aluminum chloride (VSC) was performed, the initial bond strength did not differ from the control (SBU vs SBU_VSC: p = 1.000); after TC, the bond strength to the contaminated surface was significantly lower compared to the control group (SBU* vs SBU_VSC*: p < 0.001).

For the universal adhesive PBA, a surface contamination with either VS or VSC initially had no influence on the µTBS (PBA vs PBA_VS: p = 0.292 and PBA vs PBA_VSC: p = 1.000). After TC, a significant decrease in bond strength was only detected for VSC contamination, but not for VS contamination (PBA* vs PBA_VSC*: p < 0.001 and PBA* vs PBA_VS*: p = 0.955).

Apart from PBA before aging (PBA_VS vs PBA_VSC: p = 0.424), the bond strength after contamination with aluminum chloride showed a significant decrease compared to ferric sulfate (SBU_VS vs SBU_VSC; SBU_VS* vs SBU_VSC*; PBA_VS* vs PBA_VSC*: all p<0.001, Table 3).

### Influence of Decontamination with EDTA on µTBS

Decontamination with EDTA after contamination with ferric sulfate (VS) led to significantly lower mean bond strengths before and after aging for SBU compared to the groups without decontamination (SBU_VS vs SBU_VS_EDTA; SBU_VS* vs SBU_VS_EDTA*: both p < 0.001). For VSC contamination, no significant effects were detectable (SBU_VSC vs SBU_VSC_EDTA: p = 1.000; SBU_VSC* vs SBU_VSC_EDTA*: p = 0.115).

For PBA, the EDTA decontamination had no significant influence on the µTBS, independent of the contamination (PBA_VS vs PBA_VS_EDTA: p = 1.000; PBA_VS* vs PBA_VS_EDTA*: p = 0.998; PBA_VSC vs PBA_VSC_EDTA: p = 1.000; PBA_VSC* vs PBA_VSC_EDTA*: p = 0.122).

### Influence of Aging on µTBS

Aging significantly affected the bond strength after contamination with aluminum chloride (VSC). The measured decrease in bond strength was present independent of the universal adhesives used (SBU_VSC vs SBU_VSC*: p < 0.001; PBA_VSC vs PBA_VSC*: p = 0.003).

The bond strength of the other 8 group pairs remained stable after aging. This includes the control groups, the contamination with ferric sulfate with or without decontamination by EDTA, as well as the groups contaminated with aluminum chloride and decontaminated EDTA.

### Fracture Types

**Fig 2:**
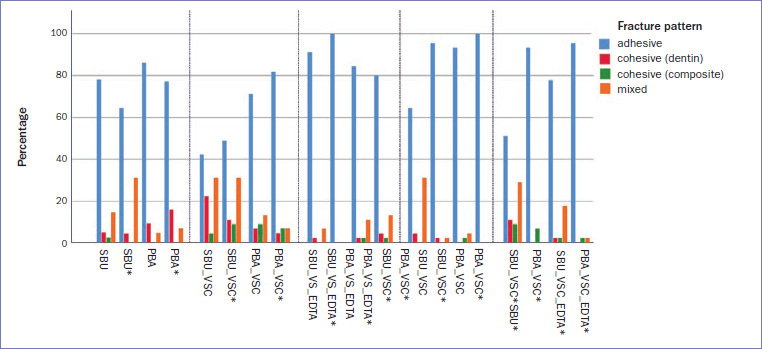
Fracture patterns of the test results (microtensile bond strength).

The chi-squared test showed significant differences between the groups (p < 0.001). Overall, the majority of fractures were adhesive (78.8%), followed by mixed fractures (12.9%) and cohesive fractures in dentin or composite (5.4%/ 2.9%). The fracture values are shown in Table 4 and the fracture patterns are illustrated in Fig 2.

**Table 4:** Results of fracture analyses before and after aging by TC (adhesive/ cohesive dentin/ cohesive composite/ mixed) in %

Fracture pattern in %								
	Adhesive	Adhesive*	Cohesive in dentin	Cohesive in dentin*	Cohesive in composite	Cohesive in composite*	Mixed	Mixed*
SBU	78.1	64.4	4.9	4.4	2.4	0.0	14.6	31.1
PBA	86.1	77.3	9.3	15.9	0.0	0.0	4.7	6.8
SBU_VS	42.2	48.9	22.2	11.1	4.4	8.9	31.1	31.1
PBA_VS	71.1	81.8	6.7	4.6	8.9	6.8	13.3	6.8
SBU_VS_EDTA	91.1	100.0	2.2	0.0	0.0	0.0	6.7	0.0
PBA_VS_EDTA	84.4	80.0	2.2	4.4	2.2	2.2	11.1	13.3
SBU_VSC	64.4	95.6	4.4	2.2	0.0	0.0	31.1	2.2
PBA_VSC	93.3	100.0	0.0	0.0	2.2	0.0	4.4	0.0
SBU_VSC_EDTA	51.1	93.3	11.1	0.0	8.9	6.7	28.9	0.0
PBA_VSC_EDTA	77.8	95.6	2.2	0.0	2.2	2.2	17.8	2.2

SBU: Scotchbond Universal; PBA: Prime & Bond Active; VS: ViscoStat (FeSO_4_); VSC: ViscoStat Clear (AlCl_3_); *after aging (TC: thermocycling).

## DISCUSSION

### Null Hypotheses

As the microtensile bond strength of SBU was significantly higher than that of PBA after aging, the first null hypothesis had to be rejected. Also, the microtensile bond strength was significantly reduced after aging and VSC contamination. Decontamination with EDTA led to a significant bond strength decrease after VS contamination and SBU application before and after aging. Regarding aging, the VSC contaminated groups showed a significant decrease in bond strength. For the rest of the control and test groups, aging did not influence the microtensile bond strength (Table 3, Fig 1). Therefore, null hypotheses 2 to 4 were partially rejected.

### Methodology

If a two-step adhesive with a self-etching primer is applied, dentin surface preparation, which creates surface roughness and leads to smear-layer formation, has a more pronounced effect on the bond strength compared to etch-and-rinse application.^26^ Sandpaper with different grain sizes creates varying conditions for bond strength: the finer the grain size, the better the bond strength of self-etch adhesives.^26^ 600-grit abrasive paper with low coarseness seems to be the most effective, as a great number of dentinal tubules appear open after its use.^26^ Therefore, 600-grit sandpaper was used for standardization in this study to create an artificial smear layer on the dentin surface, which can be compared with intraoral conditions after tooth preparation.

Due to the identical application method of the two adhesives and the same condition of the dentin, the experimental prerequisites for establishing dentin bond strength were identical. Both universal adhesives examined in the present study are “mild” adhesives due to their pH values of 2.5 (PBA) and 2.7 (SBU). In the results, it is noticeable that PBA was susceptible to aging (SBU* vs PBA*: p = 0.027), while the initial mean bond strengths of both adhesives were not significantly different (SBU vs PBA: p = 0.140), although similar tendencies were observed. Therefore, the reason for the lower long-term stability of PBA could depend on its chemical composition. As PBA is slightly more acidic than SBU, a thinner smear layer with less inorganic calcium hydroxyapatite, which forms a chemical bond with the functional monomer 10-MDP, might occur.^41^

The rather low etching potential of a mild self-etch adhesive is essentially characterized by a low demineralization depth <1 μm. This results in a very thin hybrid layer; however, in addition to the chemical bond between hydroxyapatite and 10-MDP, the micromechanical interlocking should provide sufficient bond strength.^37^ In contrast, our results partially demonstrated insufficient bond strength at the dentin-adhesive-composite interface, as reflected by the predominance of adhesive fractures (78.8%, Fig 2). This could be caused by numerous areas of remaining collagen on the dentin surface covering the dentin tubules.^37^ Nevertheless, recent evidence showed that the bond strength of 10-MDP-based self-etch adhesives and their fracture potential at the adhesive-dentin interface does not seem to be affected by its demineralizing effect.^9^ Ultimately, the formation of a three-dimensional network of self-assembled nanolayers on demineralized dentin provides mechanical strength at the interface between the adhesive and the hybrid layer, making it more resistant to biodegradation.^42^ This was partially confirmed in our study, as the groups with higher microtensile mean bond strengths tended to have a high proportion of cohesive and mixed fractures in addition to the adhesive fracture pattern, while other groups with lower mean bond strengths showed primarily adhesive failure (Tables 3 and 4; Fig 2).

### Influence of Contamination

One of the most important factors is the duration of exposure to a hemostatic agent. In the present study, the hemostatic agents were used according to the manufacturer’s specifications with regard to application and rinsing. The exposure time on the dentin surface was limited to 2 min before rinsing with a strong air-water jet for 30 s. We considered this procedure to be a clinically relevant exposure time, similar to another study.^18^ In this regard, there is wide variability in study protocols,^2,7,17,20,36^ where exposure times vary from 30 s to 5 min for ferric sulfate solutions.^17,20^

Basically, a 13.3%-15.5% ferric sulfate hemostatic agent effectively coagulates fresh blood in the gingival area.^5^ The aluminum-chloride–based hemostatic agent used (20%–25% AlCl_3_) constricts regional blood vessels, removes fluid from the tissue and causes protein precipitation.^5^ AlCl_3_ can also demineralize the dentin surface,^18^ and causes shrinkage of the gingiva and hemostasis so that the working area becomes dry and clean.^2^

The majority of all hemostatic agents, including ViscoStat and ViscoStat Clear, are water soluble. For this reason, thorough water rinsing is recommended after application of the hemostatic agent and before application of the adhesive.^8,12,13^

In contact with dentin surfaces, hemostatic agents affect their morphology. On dentin surfaces of primary teeth, ferric sulfate reduces the shear bond strength, which was caused by the coagulation of plasma proteins in the dentinal fluid.^25,28^ This is in contradiction to our results, because apparently the water solubility of ferric sulfate was great enough to not leave any potentially detrimental residues on the dentin surfaces of our samples (second dentition). After contamination with VS, the bond strength was also stable over time (SBU* vs SBU_VS*: p = 0.990; PBA vs PBA_VS: p = 0.292; PBA* vs PBA_VS*: p = 0.955). This disagreement between our results and the cited study by Prabhakar et al^28^ may have been caused by the structural differences between secondary and primary teeth (in the latter, lower mineral content, larger diameter of the dentinal tubules, thinner layer of dentin). In addition, those authors^28^ used a 15.5% ferric sulfate solution in which the specimens were immersed for 48 h without any aging procedure, as well as a different test method (shear bond strength vs μTBS) with a compressive rather than a tensile mode. In our study, the only significant effect caused by ferric sulfate was an elevated bond strength of SBU (SBU vs SBU_VS: p = 0.002); PBA showed the same tendency, but the difference was not significant (PBA vs PBA_VS: p = 0.292). Another decisive factor which may have caused contradictory results between our study and the experiments cited above, which found a significant decrease in bond strength after surface contamination with ferric sulfate, might be the use of universal adhesives. In contrast to these simplified adhesives, the two-step self-etch adhesive Clearfil SE Bond (Kuraray Noritake; Tokyo, Japan) was used with a different rinsing protocol, ie, 15-s rinsing time^28^ (our study: 30 s) or only with air drying without rinsing after application of ferric sulfate.^25^ Apart from this, the reason for the increase in bond strength might be that the hemostatic agent VS with a pH of 1.0 is very acidic,^20,39^ which causes the removal of the smear layer, opens the dentinal tubules and etches deeper into the dentin.^19,20^ The resulting surface-area enlargement might have increased the micromechanical bond compared to the control groups, in which no ferric sulfate (VS) was applied. On the other hand, this loss of smear layer and mineral content, which normally covers the dentinal tubules after dentin roughening,^18^ can be caused by both aluminum chloride and ferric sulfate solutions. Smear-layer loss can have a detrimental effect on the bonding mechanism of self-etch adhesives,^18,25^ but this was not the case in the present study, in which the bond strength increased. This surface alteration on the molecular level explains why our results after contamination with aluminum chloride (VSC) were significantly lower after aging; the creation of chemical adhesion might have been hampered, thus significantly decreasing bond strengths (SBU* vs SBU_VSC*: p < 0.001; PBA* vs PBA_VSC*: p < 0.001). Nevertheless, the use of hemostatic agents does not always reduce the bond strength,^17,30^ which also applies to the identical groups without aging (SBU vs SBU_VSC: p = 1.000; PBA vs PBA_VSC: p = 1.000). The reason for this might be that the contaminated dentin was adversely affected by aging (due to “time” and “temperature” factors), indicating reduced long-term stability.

As mentioned above, contamination with a hemostatic agent which is only air dried and not rinsed off with water drastically reduces the bond.^25^ However, this was avoided to a certain extent in our study, where 30-s rinsing was performed after contamination. Nevertheless, VSC contamination led to a decrease in bond strength, despite rinsing. Also, contamination with aluminum chloride caused a significant decrease of microtensile bond strength in direct comparison with ferric sulfate (SBU_VS vs SBU_VSC; SBU_VS* vs SBU_VSC*; PBA_VS* vs PBA_VSC*: all p < 0.001). Hence, the hemostatic agent VSC causes more severe changes in the dentin morphology, which manifests as differences in bond strength.

Decreasing mean shear bond strengths to dentin were observed after blood contamination for both two-step and all-in-one self-etch adhesives.^36^ The values of the two-step self-etch adhesive did not significantly decrease after blood contamination and application of a hemostatic agent (25% aluminum chloride), whereas the all-in-one self-etch adhesive yielded significantly lower mean bond strengths. Therefore, a two-step adhesive has been recommended for clinical use when an application of an aluminium chloride-based hemostatic agent is required to prevent blood contamination of the dentin surface.^36^ Moreover, the bond strength of contaminated surfaces can be significantly increased by a longer application time of a self-etch adhesive.^18^ All in all, the negative effect of a hemostatic agent when in contact with dentin seems to depend on the adhesive used. Self-etch adhesives react more sensitively to changes of the dentin surface than etch-and-rinse adhesives.^10^ In the clinical situation, the contamination of the dentin surface is most likely caused by a mixture of hemostatic agents, saliva, and blood, which also might alter the dentin surface and influence bond strength in a material-dependent way. Therefore, further studies should focus on different adhesives and especially combinations of hemostatic agents with blood or saliva contamination.

### Types of Decontamination

EDTA can dissolve the mineral phase of dentin, but does not denature the proteins contained in dentin and substantially change the dentin structure.^6^ EDTA as a pre-treatment agent has a positive effect on bond strength to dentin when used with self-etch adhesives.^30^ This was not specifically addressed in our study, but an application of EDTA for “cleaning” after contamination with ferric sulfate and subsequent application of Scotchbond Universal had a significantly negative effect on the mean bond strengths (SBU_VS vs SBU_VS_EDTA; SBU_VS* vs SBU_VS_EDTA*: both p < 0.001). The reason for this result could be that EDTA is a chelating agent that removes organic calcium ions from the dentin surface. This leads to reduced chemical bonding capacity, a risk of dentin erosion and reduced microhardness.^4,11^ In contrast to the present study, Dhawan et al^11^ and Baldasso et al^4^ performed their experiments on root dentin. The significant decrease in bond strength after contamination with VS and subsequent SBU application as mentioned above was also negatively influenced by the interaction with EDTA. This adverse effect was not observed for Prime & Bond Active, nor for pre-treatment with aluminum chloride and EDTA decontamination with both adhesives (PBA_VS vs PBA_VS_EDTA: p = 1.000; PBA_VS* vs PBA_VS_EDTA*: p = 0.998; PBA_VSC vs PBA_VSC_EDTA: p = 1.000; PBA_VSC* vs PBA_VSC_EDTA*: p = 0.122; SBU_VSC vs SBU_VSC_EDTA: p = 1.000; SBU_VSC* vs SBU_VSC_EDTA*: p = 0.115). It seems that the influence of EDTA decontamination on microtensile bond strength depends highly on the astringent and the adhesive used (Table 3, Fig 1). Our results do not allow a specific statement about the effect of EDTA, as no groups with solely EDTA application were examined.

Regardless of the hemostatic agent, rinsing with water to decontaminate is by far the least effective method for increasing bond strength after contamination.^7^ Despite the water solubility of hemostatic agents,^8^ larger aluminum residues remain on the dentin surface when compared to etching with phosphoric acid.^18^ Because of its low pH value of 0.5,^27^ acid etching is intended to dissolve small amounts of residue of the hemostatic agent while demineralizing the dentin.^7,34^ The disadvantage of this method is that the dentin is etched deeply, so that a self-adhesive cement cannot infiltrate the demineralized dentin entirely;^23^ the same may also apply to self-etch adhesives. Collagen which is not hybridized by the adhesive is prone to hydrolysis, leading to a reduction in bond strength.^7^ For this reason, phosphoric acid etching was not performed in this study. Also, a mechanical pre-treatment for self-etch adhesives could be considered, eg, with aluminum oxide particles (size 27 μm, at 40 psi) for removing the remains of the hemostatic agent, similar to a sandblaster. Compared to rinsing with water, this procedure has been reported to be more effective in achieving better bond strength of a self-adhesive resin cement.^7^

### Function of Aging (Thermocycling)

The mean bond strengths of adhesives can decrease by aging procedures such as thermocycling and water storage.^33^ Therefore, an experimental design as in the present study should always include such procedures in order to examine impacts on the long-term stability of adhesion.^3^ Our study demonstrated stable long-term bonding performance for the majority of groups; only the VSC-contaminated groups showed a significant decrease in bond strength (SBU_VSC vs SBU_VSC*: p < 0.001; PBA_VSC vs PBA_VSC*: p = 0.003). Therefore, within the parameters of this study, the bond strength after contamination of the dentin surface with aluminum chloride appeared less able to withstand simulated aging by thermocycling.

## CONCLUSION

Aluminum chloride decreased the microtensile bond strength of both universal adhesives (SBU and PBA) after aging. The ferric sulfate solution did not negatively affect the bond strength. In general, decontamination with EDTA with the intent to increase mean bond strengths after contamination was not effective or even had a detrimental effect on the microtensile bond strength. Clinically, dentin contamination, especially with aluminum-chloride solution, might impair the bond strength of a mild universal adhesive when used in self-etch mode and should therefore be avoided.

## Clinical relevance:

Dentin contamination by hemo-static agents, especially aluminum chloride, and decontamination with EDTA detrimentally affect the bond strength of a mild universal adhesive when used in self-etch mode and should be avoided, depending on the adhesive used.
